# Effectiveness and Safety of the Coadministration of Rifampin and Warfarin versus Direct Oral Anticoagulants: A Cohort Study

**DOI:** 10.1155/2024/9694592

**Published:** 2024-09-25

**Authors:** Ju-Chieh Wung, Chia-Chen Hsu, Chi-En Wang, Yaa-Hui Dong, Chia-Chieh Lin, Szu-Yu Wang, Shih-Lin Chang, Yuh-Lih Chang

**Affiliations:** ^1^ Department of Pharmacy Taipei Veterans General Hospital, Taipei, Taiwan; ^2^ Department of Pharmacy College of Pharmaceutical Sciences National Yang Ming Chiao Tung University, Taipei, Taiwan; ^3^ Department of Pharmacy National Taiwan University Hospital, Taipei, Taiwan; ^4^ Institute of Public Health School of Medicine National Yang Ming Chiao Tung University, Taipei, Taiwan; ^5^ Heart Rhythm Center and Division of Cardiology, Department of Medicine Taipei Veterans General Hospital, Taipei, Taiwan; ^6^ Department of Experimental Examination Healthcare and Services Center Taipei Veterans General Hospital, Taipei, Taiwan; ^7^ School of Medicine National Yang Ming Chiao Tung University, Taipei, Taiwan; ^8^ Institute of Pharmacology College of Medicine National Yang Ming Chiao Tung University, Taipei, Taiwan

## Abstract

**Introduction:**

Pharmacokinetic studies have shown that rifampin reduces the levels of oral anticoagulants during the initiation of coadministration, raising concerns about an increased thrombotic risk, but there are limited comparative clinical outcomes between rifampin and warfarin compared with direct oral anticoagulants (DOACs). This study aimed to evaluate the effectiveness and safety of concurrent use of rifampin and warfarin versus DOACs, with assessments of outcome-associated factors and oral anticoagulant (OAC) management quality.

**Methods:**

A total of 142 patients given rifampin plus warfarin (*n* = 56) or DOACs (*n* = 86) for over 7 days were included, and their clinical data and outcomes were compared.

**Results:**

The median Charlson Comorbidity Index and HAS-BLED (hypertension, abnormal renal/liver function, stroke, bleeding history or predisposition, labile INR, elderly, drugs/alcohol concomitantly) score of the two groups were 2 and 3, respectively. The incidence rate of composite ischemic or thromboembolic events was 2.16 and 1.44 per 10,000 patient-days in the warfarin and DOAC groups, respectively, with an adjusted hazard ratio (HR) of 0.41 (95% confidence interval [CI] 0.02–7.34). The incidence rate of composite major bleeding or clinically relevant nonmajor bleeding events was 1.58 and 1.52 per 10,000 patient-days in the warfarin and DOAC groups, respectively, with an adjusted HR of 1.12 (95% CI 0.32–4.45). The risk of composite bleeding events increased with a higher HAS-BLED score (HR: 1.62, 95% CI: 1.02–2.63). Moreover, 34.3% of warfarin users maintained a percent time in therapeutic range of above 50%. Furthermore, 77.9% of DOAC users received appropriate dosing.

**Conclusion:**

No significant differences were observed in terms of the incidence of thrombotic or bleeding events between the two groups during coadministration. In addition, a higher HAS-BLED score was associated with a greater risk of bleeding events regardless of the class of OACs used. Finally, close monitoring of bleeding events should be considered.

## 1. Introduction

Oral anticoagulants (OACs) include vitamin K antagonists (e.g., warfarin) and direct oral anticoagulants (DOACs) including direct thrombin inhibitor (e.g., dabigatran) and factor Xa inhibitors (e.g., apixaban, edoxaban, and rivaroxaban). OACs have been widely used in the prophylaxis and treatment of thromboembolism [1–5]. Worldwide guidelines prefer DOACs over warfarin due to noninferior or superior safety and efficacy outcomes [6, 7], but selecting the appropriate OAC can be challenging because of the limited evidence regarding effectiveness and safety in clinical scenarios involving unavoidable drug-drug interactions.

Rifampin, commonly prescribed for tuberculosis and Gram-positive infections, is a potent inducer of CYP3A4-type cytochrome P450 (CYP3A4) and P-glycoprotein (P-gp) [8]. In the presence of rifampin, warfarin (a known substrate of CYP3A4) experiences a 15%–74% decrease in its area under the curve (AUC) according to pharmacokinetic studies. Thus, a dose increase of up to 165% is required to reach a steady state in the initial 30 days of coadministration with rifampin [9, 10]. However, case studies have revealed that it could take up to 73 days after coadministration to achieve the first therapeutic international normalized ratio (INR) [11–13]. However, similar pharmacokinetic changes were observed in coadministration of rifampin and DOACs. Dabigatran is recognized as a P-gp substrate, whereas factor Xa inhibitors are recognized as substrates for both P-gp and CYP3A4. Accordingly, pharmacokinetic and case studies have reported significant decreases in the AUC or plasma levels of DOACs when coadministered with rifampin. Thus, DOACs were advised to be avoided or used with great caution in patients taking rifampin [6, 14–17]. This raises concerns about the potentially reduced drug efficacy and an increased risk of thrombosis, particularly with DOACs using fixed-dose regimens without the dose titration strategy employed with warfarin.

The choice of OACs during concurrent rifampin use involves a delicate balance between efficacy and safety. Rifampin-induced coagulopathy resulting in bleeding complications has been reported [18, 19]. Bleeding is a common concern due to the drug-drug interactions of warfarin [20]. Retrospective cohort studies have indicated a higher incidence of major bleeding among atrial fibrillation (AF) patients taking both DOACs and rifampin, compared with those exclusively on DOACs [21]. Conversely, two studies reported heterogeneous outcomes in AF patients regarding major bleeding and ischemic stroke when using rifampin with DOACs or warfarin over a 12-month follow-up [22, 23], but detailed information was lacking regarding the OAC treatment qualities. Moreover, the clinical management strategies can address not only major bleeding but also clinically relevant nonmajor bleeding (CRNMB). Thus, we conducted a retrospective observational cohort study to evaluate the effectiveness and safety of OACs and rifampin coadministration, incorporating an analysis of OAC treatment qualities and a characterization of factors associated with bleeding events.

## 2. Methods

### 2.1. Institutional Review Board

This study was approved by the Institutional Review Board (IRB) of Taipei Veterans General Hospital (TPEVGH IRB; no. 2022-05-014BC) and according to the principles of the Declaration of Helsinki. Because the investigation posed a minimal risk to the participants and did not involve procedures, the requirement for written informed consent from the patients was waived by the TPEVGH IRB.

### 2.2. Study Design

This retrospective observational cohort study was conducted in a large academic medical center in northern Taiwan. TPEVGH annually caters to more than 2 million outpatient visits for 1.1 million outpatients and 3000 beds for 110,000 inpatients. On average, around 25,000 drug prescriptions are generated daily for ambulatory patients.

### 2.3. Participants

**Figure 1:**
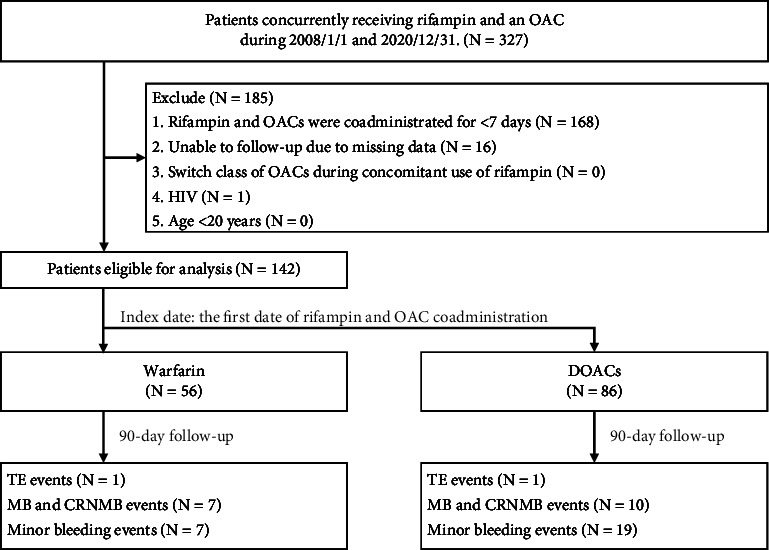
Study inclusion flowchart. CRNMB, clinically relevant nonmajor bleeding; DOACs, direct oral anticoagulants; HIV, human immunodeficiency virus; MB, major bleeding; OACs, oral anticoagulants; TE, thromboembolic event.

This study included patients concurrently receiving rifampin and an OAC during January 1st, 2008, and December 31st, 2020 in TPEVGH. We excluded patients in whom rifampin and OACs were coadministered for less than 7 days, those who were unable to follow-up due to missing data, those who switched class of OACs during concomitant use of rifampin, those with human immunodeficiency virus (HIV), and those aged <20 years (Figure 1). Patients with HIV infection and those younger than 20 years old were excluded to mitigate potential bias in our study. This is also consistent with the ethical and regulatory considerations mandated by the IRB due to concerns regarding confidentiality and the need for increased protection of this vulnerable population.

### 2.4. Data Sources/Measurements/Variables

Patient characteristics, including demographics, comorbidities, medical records, laboratory data, and concomitant medications, were retrieved from the electronic medical records and hospital information system. Comorbidities were defined using International Classification of Diseases (ICD) codes diagnosed at least twice within 1 year, using either ICD, Ninth Revision, Clinical Modification or ICD-10-CM. Diabetes mellitus and hypertension were classified based on the ICD code and their corresponding prescription data. Heart failure was defined using the ICD code with one of the following criteria: left ventricular ejection fraction of <40%, admission with ICD code of acute decompensated heart failure within 1 year, brain natriuretic peptide (BNP) > 35 pg/ml or N-terminal probrain natriuretic peptide (NT-pro-BNP) ≥125 pg/ml, or currently under one of the heart failure guideline-directed medical therapies. Malignancy was defined using an ICD code with active status; patients were classified as receiving cancer treatment within 6 months prior to the index date. Percent time in therapeutic range (TTR%) was calculated using an open-source Excel sheet under the principle of the Rosendaal method downloaded from https://www.inrpro.com/rosendaal.asp [24]. Dosing guidance for DOACs was obtained from the package insert and Lexicomp Online [25]. DOAC regimens were categorized as follows: appropriate standard dose, appropriate reduced dose, overdosing, and underdosing. Inappropriate regimens, such as overdosing and underdosing, were considered deviations from the recommended dose. Overdosing was defined as prescribing the standard dose of a DOAC despite meeting the criteria for dose reduction, whereas underdosing was defined as prescribing a reduced dose of a DOAC despite not meeting the criteria for dose reduction [26].

Patients were divided into either the warfarin group or DOAC group and were followed up to 90 days. The index date was defined as the first date of rifampin and OAC coadministration. The primary outcomes included the incidence rates and hazard ratio (HR) of composite ischemic or thromboembolic events, as well as those of composite major bleeding or CRNMB events. The secondary outcomes included the incidence rates and HR of major bleeding events, CRNMB events, and minor bleeding events. Ischemic and thromboembolic events (e.g., ischemic stroke, systemic embolism, venous thrombosis, and acute myocardial infarction) were identified by ICD code combined with diagnostic imaging reports and relevant laboratory data. Major bleeding events and CRNMB events were characterized using the definition of International Society on Thrombosis and Haemostasis [27]. All reported bleeding not classified as major bleeding or CRNMB events were categorized as minor bleeding events. All events were independently identified by two authors. The two authors discussed any discrepancies to arrive at a consensus or consulted with another author to reach a final decision. Follow-up was defined from the index date to the occurrence of the outcome event, death, loss of follow-up, or the end of 90-day follow-up, whichever came first.

### 2.5. Statistical Methods

Descriptive statistics were used to summarize the characteristics of the patients. None of the continuous variables were normally distributed, as examined by the Shapiro–Wilk test and visual inspection of the histograms. The continuous variables are presented as medians (interquartile ranges, IQR). Categorical variables were described as frequencies and percentages. We compared baseline characteristics between the two groups using the Mann–Whitney *U* test and Fisher's exact test for continuous and categorical variables, respectively. Cox proportional hazards models with a Firth correction for rare outcomes were used to estimate HR and 95% confidence intervals (CIs) for outcomes comparing the warfarin group with the DOAC group. Furthermore, factors associated with the primary outcomes were also examined. Statistical analyses were performed using the Statistics Analysis System software version 9.4 (SAS Institute Inc., Cary, NC, USA), with two-sided *P* values of <0.05 considered statistically significant.

## 3. Results

### 3.1. Participants and Descriptive Data

We detected the concurrent use of rifampin and an OAC in a cohort of 327 patients from January 1, 2008, to December 31, 2020. After excluding patients with combined rifampin and an OAC for less than 7 days, those lost to follow-up due to missing data, and those with HIV infection, 142 patients were included. The warfarin group included 56 patients, whereas the DOAC group included 86 patients, among whom 17 (19.8%), 14 (16.3%), 16 (18.6%), and 39 (45.3%) were prescribed dabigatran, apixaban, edoxaban, and rivaroxaban, respectively. The median age of patients in the warfarin group was 80.4 (69.3–84.2) years, with 47 (83.9%) males. In the DOAC group, the median age was 86.2 (78.6–89.9) years, with 66 (76.7%) males. In both groups, AF was the leading indication for OAC use, seen in 46.4% and 90.7% in the warfarin and DOAC groups, respectively, being notably higher in the latter. In the warfarin group, venous thromboembolism was the second most common indication at 32.1%. The higher prevalence of AF as an indication for DOAC usage led to an increased percentage of amiodarone/dronedarone and diltiazem/verapamil administration, with respective rates of 17.4% and 33.7% in the DOAC group and 5.4% and 16.1% in the warfarin group. The median CHA_2_DS_2_-VASc [congestive heart failure, hypertension, age ≥75 years (doubled), diabetes mellitus, prior stroke or TIA or thromboembolism (doubled), vascular disease, age 65 to 74 years, sex category-female] score of patients with AF in both groups was 4. As caution is taken when prescribing DOACs to patients undergoing dialysis, only 1.2% of the DOAC group had renal impairment (i.e., with a history of dialysis or estimated creatinine clearance <15 mL/min), in contrast to 23.2% in the warfarin group. Patients in the warfarin group, versus the DOACs group, had a lower median hemoglobin level (10.6 [9.5–12.0] vs. 11.4 [9.4–11.4] g/dL) and platelet count (180 [131–267] × 10^3^ vs. 224 [157–284] × 10^3^/mcL). The median Charlson Comorbidity Index was 2 (1–3) in the warfarin group and 2 (0–3) in the DOAC group. The median HAS-BLED (hypertension, abnormal renal/liver function, stroke, bleeding history or predisposition, labile INR, elderly, drugs/alcohol concomitantly) score of the two groups was 3 (2–4) (Table 1).

In the warfarin group, 32 out of 56 patients were using warfarin before the coadministration of rifampin. In the 180 days preceding the index date, 6 (18.7%), 5 (15.6%), and 14 (43.8%) patients achieved TTR% values above 70%, within 50%–70%, and below 50%, respectively. Moreover, 7 (21.9%) patients had fewer than 2 INR checks within 180 days. In the DOAC group (*n* = 86), appropriate dosing was seen in 82.4% (14/17), 71.4% (10/14), 75% (12/16), and 79.5% (31/39) of patients taking dabigatran, apixaban, edoxaban, and rivaroxaban, respectively (Table 2). The major appropriate dosing type was reduced dose (58/67), while the major inappropriate dosing type was underdosing (15/19). There were 71 out of 86 patients using DOACs prior to the coadministration of rifampin.

### 3.2. Primary and Secondary Outcomes

The incidence of composite ischemic or thromboembolic events was 2.16 and 1.44 per 10,000 patient-days in the warfarin and DOAC groups, respectively, with a crude HR of 0.67 (0.06–8.27) and an adjusted HR of 0.41 (95% CI: 0.02–7.34) (Table 3). For composite major bleeding or CRNMB events, the incidence was 1.58 and 1.52 per 10,000 patient-days in the warfarin and DOAC groups, respectively, yielding a crude HR of 0.95 (0.37–2.52) and an adjusted HR of 1.12 (95% CI 0.32–4.45). The risk of composite major bleeding or CRNMB events increased with a higher HAS-BLED score (HR: 1.62, 95% CI: 1.02–2.63) (Table 4). Meanwhile, major bleeding events occurred at an incidence of 4.33 and 8.88 per 10,000 patient-days in the warfarin and DOAC groups, respectively, resulting in a crude HR of 1.77 (0.45–9.63) and an adjusted HR of 2.77 (95% CI 0.44–21.69). CRNMB events had an incidence of 11.21 and 5.86 per 10,000 patient-days in the warfarin and DOAC groups, respectively, with a crude HR of 0.54 (0.15–1.90) and an adjusted HR of 0.52 (95% CI 0.08–3.49). Minor bleeding events were observed at an incidence of 14.45 and 27.50 per 10,000 patient-days in the warfarin and DOAC groups, respectively, with a crude HR of 1.82 (0.82–4.51) and an adjusted HR of 1.73 (95% CI 0.62–5.40). None of these outcomes were significantly different between the two groups.

## 4. Discussion

This observational cohort study found no statistically significant differences in the incidence rates and HR for composite ischemic or thromboembolic events and composite major bleeding or CRNMB events between patients under the initial concurrent use of rifampin with warfarin versus DOACs, with warfarin having a suboptimal management quality than DOACs. In addition, a higher HAS-BLED score was associated with a greater risk of bleeding events regardless of the class of OACs used.

Our analysis of warfarin management quality before the coadministration of rifampin showed that 34.3% of patients maintained a TTR% above 50%, indicating suboptimal management. This finding was similar to another observational study in the same region, wherein 21% of AF patients maintained a TTR% above 50% [28]. Warfarin use was associated with a lower TTR% in the Chinese population [29]. Diabetes mellitus, ischemic heart disease, and peripheral vascular disease were associated with a worse INR stability, with the first two comorbidities accounting for more than a quarter each in our population. Additionally, previous studies have revealed that genetic polymorphisms in CYP2C9 (cytochrome P450 2C9) and VKORC1 (vitamin K epoxide reductase complex 1) are correlated with warfarin dosage requirements and the time required to achieve therapeutic INR levels [30]. Warfarin management guided by pharmacogenetic tests is associated with a higher TTR% compared with standard care without genetic testing [31, 32]. However, pharmacogenetic tests of these variants were not available during the study period (2008–2020), thereby potentially contributing to suboptimal warfarin management. However, 77.9% of DOAC users were receiving the appropriate regimen. The incidence rates and HR of composite ischemic or thromboembolic events did not differ based on the anticipated decrease of DOAC levels after coadministration. Our findings could provide valuable insights for optimizing anticoagulation strategies in this patient population.

In our study, the observed incidence rates of major bleeding in the warfarin and DOAC groups were 4.33 and 8.88 per 10,000 patient-days, respectively. These rates exceeded those reported in a national cohort, documented at 2.11 and 5.67 per 1000 person-years, equivalent to 0.06 and 0.16 per 10,000 patient-days, respectively [23]. Based on the subgroup analyses, a high bleeding risk was more associated with DOAC than warfarin in patients with a HAS-BLED score of 0–2. However, no differences were observed among patients with a HAS-BLED score of ≥3 [23]. Compared with the cohorts in other studies, our population, including patients with end-stage renal disease and OAC indications other than AF, was older and had a higher HAS-BLED score [22, 23]. Results showed that the risk of bleeding events increased with a higher HAS-BLED score regardless of the type of OACs used. Thus, there is a need for close monitoring and management of bleeding when coadministering OACs and rifampin in patients with higher HAS-BLED scores, regardless of the class of OACs used.

This study first assessed the effectiveness and safety of initiating the coadministration of rifampin with warfarin and DOACs. Additionally, our study first reported laboratory data associated with the characteristics of the patients, including renal and liver function, blood test findings, use sequence of OACs and rifampin, and management quality of OACs. However, the current study had several limitations. First, the small sample size and low incidence of some outcomes in this study resulted in a wider 95% confidence interval for the corresponding hazard ratio point estimates. This phenomenon might have reduced the precision and confidence of the results. Small sample sizes can result in a reduced statistical power, which increases the risk of type II error. Thus, the study could have failed to detect a true effect. A small sample size may also reduce the precision of the point estimates and increase the effect of random error on the results. Hence, studies with a larger sample size should be performed to validate our findings. Second, we could not control or collect all confounding factors in this study due to its retrospective design. Third, the scale of single-center studies limits generalizability, and the sample size is insufficient for subgroup analysis. Larger scale studies are essential to provide validated evidence, not only for rifampin initiation but also for its discontinuation when coadministered with OACs.

## 5. Conclusion

During the initiation of concurrent use of rifampin with warfarin compared with DOACs, there were no significant differences in thrombotic and bleeding risks. However, patients in our cohort with a higher HAS-BLED score showed a higher risk of bleeding events regardless of the class of OACs used; close monitoring for unwanted bleeding events should be considered.

## Figures and Tables

**Table 1:** Patient characteristics.

Variable	Warfarin (*N* = 56)	DOACs (*N* = 86)	*P* value
Age (yr)	80.4 (69.3–84.2)	86.2 (78.6–89.9)	**<0.001**
Sex, male, *n* (%)	47 (83.9)	66 (76.7)	0.395
BMI (kg/m^2^)	22.3 (19.6–26.8)	20.8 (19.6–23.5)	0.118
CHA_2_DS_2_-VASc score^†^	4 (4–5)	4 (3–5)	
HAS-BLED score	3 (2–4)	3 (2–4)	0.350
OAC use first, *n* (%)	32 (57.1)	71 (82.6)	**<0.001**
Indication of OAC, *n* (%)			**<0.001**
Atrial fibrillation	26 (46.4)	78 (90.7)	
Venous thromboembolism	18 (32.1)	7 (8.1)	
Others	12 (21.4)	1 (1.2)	
Indication of rifampin, *n* (%)			0.269
Tuberculosis	43 (76.8)	73 (84.9)	
Others	13 (23.2)	13 (15.1)	
Laboratory data			
CrCl (ml/min)	32.5 (17.9–57.5)	42.5 (33.3–55.6)	0.090
ALT (U/L)	17.0 (12.0–31.5)	16.5 (12.0–23.0)	0.330
AST (U/L)	24.0 (18.0–36.0)	25.0 (20.0–31.0)	0.776
Hemoglobin (g/dL)	10.6 (9.5–12.0)	11.4 (9.4–11.4)	**0.005**
Platelet (10^3^/*µ*L)	180 (131–267)	224 (157–284)	**0.023**
INR	1.13 (1.07–1.41)	1.15 (1.05–1.27)	0.516
Quan-CCI	2 (1–3)	2 (0–3)	0.735
Comorbidities, *n* (%)			
Hypertension	36 (64.3)	63 (73.3)	0.268
Heart failure	20 (35.7)	28 (32.6)	0.720
Diabetes mellitus	17 (30.4)	22 (25.6)	0.567
Ischemic heart disease	15 (26.8)	27 (31.4)	0.579
Stroke	12 (21.4)	13 (15.1)	0.372
Cancer	6 (10.7)	15 (17.4)	0.337
Peripheral vascular disease	2 (3.6)	4 (4.7)	1
Systemic embolism	2 (3.6)	2 (2.3)	0.647
Dialysis/CrCl <15 mL/min	13 (23.2)	1 (1.2)	**<0.001**
Medications, *n* (%)			
NSAID	4 (7.1)	4 (4.7)	0.712
Antiplatelet	11 (19.6)	9 (10.5)	0.143
Digoxin	7 (12.5)	10 (11.6)	1
H_2_ blocker/PPI	22 (39.3)	28 (32.6)	0.473
Amiodarone/dronedarone	3 (5.4)	15 (17.4)	**0.040**
Diltiazem/verapamil	9 (16.1)	29 (33.7)	**0.021**

^†^Calculated only for patients with atrial fibrillation. Data are presented as the median (IQR) if not stated. ALT, alanine aminotransferase; AST, aspartate aminotransferase; BMI, body mass index; CCI, Charlson Comorbidity Index; CHA_2_DS_2_-VASc, congestive heart failure, hypertension, age ≥75 years (doubled), diabetes mellitus, prior stroke or TIA or thromboembolism (doubled), vascular disease, age 65 to 74 years, sex category-female; CrCl, creatinine clearance; DOACs, direct oral anticoagulants; HAS-BLED, hypertension, abnormal renal/liver function, stroke, bleeding history or predisposition, labile INR, elderly, drugs/alcohol concomitantly; INR, international normalized ratio; IQR, interquartile range; NSAID, nonsteroidal anti-inflammatory drug; OAC, oral anticoagulant; PPI, proton pump inhibitor. The bold values indicate the variables in the two groups show statistically difference.

**Table 2:** Analysis of DOAC dosing appropriateness.

DOACs number (%)	Dabigatran *N* = 17	Apixaban *N* = 14	Edoxaban *N* = 16	Rivaroxaban *N* = 39	Total *N* = 86
Appropriate	**14 (82.4)**	**10 (71.4)**	**12 (75.0)**	**31 (79.5)**	**67 (77.9%)**
Standard dose	1	1	0	7	9
Reduced dose	13	9	12	24	58

Inappropriate	**3 (17.6)**	**4 (28.6)**	**4 (25.0)**	**8 (20.5)**	**19 (22.1%)**
Overdosing	2	0	1	1	4
Underdosing	1	4	3	7	15

DOACs, direct oral anticoagulants. The bold values are the sum of number of standard dose and reduced dose, and the sum of number of overdosing and underdosing, respectively.

**Table 3:** Event rates and hazard ratios of the outcomes comparing warfarin versus DOAC, in combination with rifampin.

	Warfarin group *N* = 56			DOACs group *N* = 86			Crude HR (95% CI)	aHR (95% CI)
	Mean follow-up days	Events	Incidence per 10,000 PD	Mean follow-up days	Events	Incidence per 10,000 PD		
Primary outcomes								
Composite ischemic or thromboembolic events	82.6	1	2.16	81.0	1	1.44	0.67 (0.06–8.27)	0.41 (0.02–7.34)^a^
Composite MB or CRNMB events	79.2	7	1.58	76.4	10	1.52	0.95 (0.37–2.52)	1.12 (0.32–4.45)^b^
Secondary outcomes								
MB events	82.4	2	4.33	78.6	6	8.88	1.77 (0.45–9.63)	2.77 (0.44–21.69)^b^
CRNMB events	79.6	5	11.21	79.3	4	5.86	0.54 (0.15–1.90)	0.52 (0.08–3.49)^b^
Minor bleeding events	86.5	7	14.45	80.3	19	27.50	1.82 (0.82–4.51)	1.73 (0.62–5.40)^b^

^a^Adjusted for age and sex, ^b^Adjusted with age, sex, HAS-BLED score, OAC use first, indication of OAC, dialysis/CrCl <15 mg/dL, hemoglobin, platelet, use of amiodarone/dronedarone, use of diltiazem/verapamil. CI, confidence interval; CRNMB, clinically relevant nonmajor bleeding; DOACs, direct oral anticoagulants; HR, hazard ratio; MB, major bleeding; PD, patient-days.

**Table 4:** Factors associated with risk of composite major bleeding and CRNMB outcomes in patients receiving warfarin versus those receiving DOAC, in combination with rifampin (*n* = 142).

	Adjusted hazard ratio	95% CI	*P* value
Age, years^†^	1.05	0.99–1.13	0.866
Sex			
Male	Ref		
Female	2.50	0.75–7.70	0.137
HAS-BLED score^†^	**1.62**	**1.02**–**2.63**	**0.048**
Indication of OAC			
Atrial fibrillation	Ref		
Venous thromboembolism	2.61	0.50–12.52	0.256
Others	2.92	0.44–15.37	0.253
Dialysis treatment/CrCl <15 mg/min	1.70	0.36–7.36	0.499
Hemoglobin level (g/dL^†^)	0.85	0.59–1.19	0.363
Platelet count, (10^3^/*µ*L^†^)	1.00	1.00–1.00	0.551
Use of amiodarone/dronedarone	4.12	0.89–17.13	0.068
Use of diltiazem/verapamil	1.80	0.53–5.66	0.347
OAC use before the index date	1.12	0.29–5.37	0.885

^†^Each 1-unit increase, CI, confidence interval; CRNMB, clinically relevant nonmajor bleeding; CrCl, creatinine clearance; DOACs, direct oral anticoagulants; HAS-BLED, hypertension, abnormal renal/liver function, stroke, bleeding history or predisposition, labile INR, elderly, drugs/alcohol concomitantly; OAC, oral anticoagulant. The bold values indicate a higher HAS-BLED score was associated with a greater risk of bleeding events.

## Data Availability

The data presented in this research are in accordance with the regulations of the IRB of TPEVGH. These data are restricted for research purposes only and can be made available upon request from the corresponding author or the IRB of TPEVGH (e-mail: irbopinion@vghtpe.gov.tw).
